# Arabic validation of the Compulsive Internet Use Scale (CIUS)

**DOI:** 10.1186/1747-597X-6-32

**Published:** 2011-11-29

**Authors:** Yasser Khazaal, Anne Chatton, Khodor Atwi, Daniele Zullino, Riaz Khan, Joël Billieux

**Affiliations:** 1 Department of Mental Health and Psychiatry, Geneva University Hospitals, Geneva, Switzerland; 2 Islamic University, Khaldeh, Lebanon; 3 Psychological Sciences Research Institute. Catholic University of Louvain-La-Neuve, Belgium

## Abstract

**Background:**

The popularity of using the Internet and related applications has grown in Arabic countries in recent years. Despite numerous advantages in terms of optimizing communications among individuals and social systems, the use of the Internet may in certain cases become problematic and engender negative consequences in daily life. As no instrument in the Arabic language is available, however, to measure excessive Internet use, the goal of the current study was to validate an Arabic version of the Compulsive Internet Use Scale (CIUS).

**Methods:**

The Arabic version of the CIUS was administered to a sample of 185 Internet users and exploratory and confirmatory analyses performed.

**Results:**

As found previously for the original version, a one-factor model of the CIUS had good psychometric properties and fit the data well. The total score on the CIUS was positively associated with time spent online.

**Conclusion:**

The Arabic version of the CIUS seems to be a valid self-report to measure problematic Internet use.

## Background

The prevalence of Internet use has increased worldwide during the last decade. Although this provides wonderful opportunities for communication, exchange, and social interactions, it has been accompanied by the development, in some individuals, of an excessive and noncontrollable pattern of use (i.e., problems related to Internet use, difficulty stopping, continuing use despite the intention to stop), leading to the emergence of the concept of Internet addiction [1-4].

The proposed diagnostic criteria [5,6] include obsessive thoughts regarding the Internet, loss of control (Internet usage more than intended or despite the negative consequences), withdrawal symptoms, and tolerance. Internet-related activities and preoccupations disturb major aspects of real life such as time management, sexual life and marriage, work, and academic and financial activities [7,8]. The phenomenon has been associated with several psychiatric disorders and symptoms [9], such as depression and social phobia [10], impulsivity [11,12], and substance misuse [13]. Furthermore, studies carried out on a population with a large age range showed that younger Internet users were at increased risk for Internet addiction [14,15]. In addition, male gender was possibly linked to Internet addiction [15-18]. This phenomenon was postulated to be linked to intermediate psychosocial variables, such as more "escapism" (avoidance) [11] in males than in females, or to differences related to the Internet applications used, such as online games [19].

Nowadays, there are roughly 65.4 million Internet users in the Arab world, representing only 18.9% of about 347 million persons [20]. Between 2000 and 2010, the growth of Internet use in Arab countries was the highest among the top 10 Internet users by language, or 2500% in comparison to 281% for English-language countries [20]. The Internet seems, furthermore, to play an important role in social changes occurring in the Arab world. Internet-related applications such as Facebook or Twitter have recently played a key role in the Arab street revolutions in 2011, referred to as "Arabic spring."

Unfortunately, despite the growing importance of Internet use in Arabic-speaking countries, there is, to the best of our knowledge, no validated instrument to measure problematic Internet use in Arab-speaking samples. In recent years, several instruments have been developed to assess problematic Internet use, such as Young's questionnaire and the Internet Addiction Test [8,21]. Some of these instruments identify the presence or absence of specific diagnostic criteria (with yes or no questions), diminishing the ability to measure the phenomenon in a more continuous manner from nonproblematic Internet use to its more severe forms. Furthermore, several scales, such as the Internet Addiction Test [21], include items related to psychosocial aspects of Internet use rather than to Internet addiction-related core constructs (e.g., Item 4: Do you form new relationships with fellow online users?). For Meerkerk et al. [22], it seems then necessary to validate, on a large sample, a short questionnaire that measures on its continuum (with Likert scales) the severity of specific core problematic Internet use symptoms. From this perspective, they developed a 14-item Compulsive Internet Use Scale (CIUS) that provides a dimensional score (i.e., severity) of problematic Internet use. The questionnaire covers a number of core components of addictive behaviors such as loss of control, preoccupation, withdrawal symptoms, salience, conflict, and coping (i.e., use of the Internet as an escape strategy). Positive correlations were reported between the amount of time spent online, self-reported problems concerning Internet use, and CIUS scores [22]. Furthermore, high correlations were found with the Online Cognition Scale [23], in particular with the subscale called diminished impulse control [22]. The CIUS was found to have high internal consistency and test-retest validity. Its structure, explored in multiple independent samples, is unifactorial [22]. The CIUS has a number of advantages over other instruments, such as its shortness, which makes it easier to use for screening in clinical settings and in online studies, and its unidimensional structure [22]. Another advantage of the CIUS is that it measures Internet uncontrolled use rather than related psychosocial well-being concepts [22].

Several versions (14 to 17 items) of the CIUS [24,25] were presented in papers accepted or published before the publication of the final validated 14-item version [22]. Furthermore, a version in which some items were modified (e.g., going online at the expense of schoolwork) was also proposed for a sample of 10- to 16-year-old school students [25]. The main goal of the present paper was to investigate the psychometric properties of the Arab-language translation of the 14-item CIUS [22] in a sample of people ≥ 15 years of age.

## Materials and methods

### Participants

A total of 186 participants took part in the study (51% females). Participants were volunteers from the community recruited through local advertising. The ages of the participants ranged from 15 to 25 years (*M *= 17.6, *SD *= 2.9).

### Instruments and procedure

All participants were screened alone using the Arab-language version of the CIUS. Participants also had to respond to three supplementary questions: (1) how much time they spent online daily, (2) whether they had an Internet connection at home, and (3) for how many years they had used the Internet.

The Arab-language adaptation of the CIUS (Additional File 1) consisted of the 14 original items [22] translated into Arabic using a translation and a back-translation procedure. All items were scored on a 5-point Likert scale: 0 (*never*); 1 (*seldom*); 2 (*sometimes*); 3 (*often*); 4 (*very often*). The translation was carried out as follows: A professional translator translated the items from English to the Arab language. Following this stage, YK and AK (two bilingual authors) translated the items into English. Discrepancies emerging between the English version and the back-translated version were discussed, and adjustments were consensually made. Finally, the scale was given to a small sample of 10 laypersons in order to check how well the translated items were understood. Because of some difficulties related to the understanding of Item 9 (negative formulation: Have you unsuccessfully tried to spend less time on the Internet?), a positive formulation was chosen for the Arab translation of the CIUS (Have you successfully tried to spend less time on the Internet?), leading to a reverse scoring of this item.

### Analyses

In this study, SPSS 18.0 for Windows and AMOS 19.0 (Analysis of Moment Structures; SPSS Inc., Chicago, IL) software programs were used to perform the statistical analyses.

First, descriptive statistics were computed for the demographic characteristics. Second, internal consistency, that is, the extent to which the CIUS items were interrelated, was measured by using Cronbach's coefficient. As this coefficient varies between 0 and 1, it may translate to a greater degree of homogeneity of the items if its value is close to 1. Too high an alpha value may signal a high level of item redundancy, that is, a number of items asking the same question in slightly different ways. Consequently, we considered an acceptable alpha value to be more than 0.70, but not much higher than 0.90 [26].

Third, the factorial structure of the CIUS was considered. The number of factors to extract was determined by an exploratory factor analysis with Velicer's minimum average partial (MAP) test performed on the correlation matrix [27,28]. The extraction method used was principal components. The number of factors suggested by the MAP test was then confirmed through parallel analysis. In parallel analysis, the focus is on the number of components that account for more variance than the components derived from random data, whereas in the MAP test, the focus is on the relative amounts of systematic and unsystematic variance remaining in a correlation matrix after extractions of an increasing number of components [28]. Both procedures are statistically based, rather than being mechanical rules of thumb. To explore the fitness of using the CIUS instrument for assessing Internet addiction, we next applied confirmatory factor analysis (CFA) in a structural equation model with AMOS software. To assess the adequacy of the model, we used a few fit measures as outlined below:

a. The χ^2 ^to degrees of freedom ratio. Several researchers have recommended the use of this ratio as a measure of fit to overcome problems associated with the χ^2 ^test statistic. These problems include, among others, violation of assumptions, model complexity, and dependence on sample size. Ratios as low as 2 seem to indicate a reasonable fit [29].

b. The root mean square error of approximation (RMSEA). This is a measure of approximate fit in the population and is therefore concerned with the discrepancy due to approximation. The RMSEA is bounded below 0. RMSEA values less than or equal to 0.05 can be considered as a good fit, between 0.05 and 0.08 an acceptable fit, and greater than 0.8 a mediocre fit, whereas values > 0.10 are not acceptable [30].

c. The comparative fit index (CFI). The CFI ranges from 0 to 1, with higher values indicating better fit. A rule of thumb is that values greater than 0.95 may be interpreted as a good fit, whereas values between 0.90 and 0.95 are indicative of acceptable fit relative to the independence model [31].

d. The standardized root mean square residual (SRMR). This is a measure of the mean absolute correlation residual, that is, the overall difference between the observed and predicted correlations. Values of the SRMR less than 0.10 are generally considered favorable [32].

While fitting a structural equation model (SEM), we are interested not only in knowing whether the model is identified, but also in the quality of the model fit. A model is said to fit the observed data to the extent that the model-implied covariance matrix is equivalent to the empirical one, minimizing the discrepancy function. In this regard, standardized residual covariance drives all tests of overall fit [33]. Standardized residuals are fitted residuals divided by their asymptotically standard errors. When they are too large, it is a sign of model misspecification. Different cutoffs are provided. For Jöreskog and Sörbom [34], values > 2.58 are considered to be large, whereas other authors [35] include the value of 1.96 in the range of values suggestive of a lack of fit. We used the cutoff of 1.96 to ascertain misspecification and to relax some constraints on the correlation measurement errors, as suggested by other authors[35].

Even though data are frequently obtained as if they were from a single population, a closer examination of the subjects might uncover population heterogeneity that rules out a single population [36]. It is then advisable to conduct a multi-sample structural model (MSSM) to ascertain the validity and the viability of the tool in different subsamples. Unfortunately, it has not been possible to conduct an MSSM, as large sample sizes and multivariate normality assumptions in each subsample are absolutely crucial for stable variances and covariances. Therefore, we resorted to a multiple linear regression with the aim of emphasizing the validity and the viability of the instrument as an MSSM.

A multiple linear regression analysis was then performed to describe the relationship between Internet addiction as the dependent variable and daily hours spent online and years of Internet use as the independent variables, after controlling for age and sex. Prior to the linear regression process, the variable "years of Internet use," a four-level categorical variable (1: less than 1 year; 2: 1 to 3 years; 3: 3 to 5 years; 4: more than 5 years) was transformed into three dichotomous variables taking on a value of 0 or 1.

The presence of outliers, that is, data points with z-scores around 3 in absolute value for large data sets or around 2.5 for a small sample size [37], was also determined. As most researchers [38] suggest, we transformed key variables in lognormal or quadratic terms to remedy this problem.

## Results

### Sample characteristics

Of the original 185 observations initially recorded, 30 were excluded because of missing data, hence leaving a final sample size of 155 participants. The time daily spent online ranged from 20 to 720 minutes per day (*M *= 150, *SD *= 108). Only 41.5% of the participants had a computer at home. The time that they had been using the Internet was distributed as follows: less than 1 year, 14.7%; between 1 and 3 years, 26.1%; between 3 and 5 years, 37.5%; and more than 5 years, 21.7%. The characteristics of the participants are reported in Table 1.

**Table 1:** Participants' Characteristics

Age, y, *M *(*SD*)	17.6 (2.9)
Female, %	51
Internet at home, %	41.5
Hours spent daily on the Internet, *M *(*SD*)	2.5 (1.8)
CIUS total score, M (SD)	22 (8.6)
Internet use for (%):	
- less than 1 year	14.7
- 1 to 3 years	26.1
- 3 to 5 years	37.5
- 5 years or more	21.7

### Internal consistency and factor structure

The internal consistency, measured by Cronbach's coefficient, was satisfactory (α = 0.78). Exploratory factor analysis by Velicer's MAP test suggested a one-factor solution. This finding was successfully confirmed through parallel analysis. This one-factor model was then evaluated by CFA. The χ^2 ^to degrees of freedom ratio was equal to 2.13, indicating a discrepancy between the data and the hypothetical model. Furthermore, the other CFA statistics, such as RMSEA (0.09) and CFI (0.78), did not meet goodness-of-fit criteria (see Table 2). Inspection of the standardized residual covariances revealed six that were beyond the value of 1.96. Hence, a new model was specified, including, among others, the correlation of variance errors of Items 4 and 7 (the Internet is the preferred activity), Items 4 and 11 (the Internet as a favorite activity and neglecting obligations), Items 5 and 7 (problem with stopping use of the Internet and looking forward to using the Internet), Items 5 and 11 (neglecting obligations), Items 8 and 9 (using the Internet less often), and Items 12 and 13 (escape), which resulted in acceptable goodness-of-fit measures, as shown in Table 2. Furthermore, all new standardized residuals fell below the 1.96 cutoff.

**Table 2:** Factor Loadings and Goodness-of-Fit Measures

*Item*	*Question*	*Model 1*	*Model 2*
Item 1	Do you find it difficult to stop using the Internet when you are online?	0.58	0.58
Item 2	Do you continue to use the Internet despite your intention to stop?	0.47	0.47
Item 3	Do others (e.g., partner, children, parents) say you should use the Internet less?	0.37	0.37
Item 4	Do you prefer to use the Internet instead of spending time with others (e.g., partner, children, parents)?	0.51	0.51
Item 5	Are you short of sleep because of the Internet?	0.51	0.51
Item 6	Do you think about the Internet, even when not online?	0.58	0.57
Item 7	Do you look forward to your next Internet session?	0.46	0.47
Item 8	Do you think you should use the Internet less often?	0.10	0.12
Item 9	Have you unsuccessfully tried to spend less time on the Internet?	0.24	0.24
Item 10	Do you rush through your (home) work in order to go on the Internet?	0.60	0.61
Item 11	Do you neglect your daily obligations (work, school, or family life) because you prefer to go on the Internet?	0.48	0.46
Item 12	Do you go on the Internet when you are feeling down?	0.41	0.38
Item 13	Do you use the Internet to escape from your sorrows or get relief from negative feelings?	0.45	0.43
Item 14	Do you feel restless, frustrated, or irritated when you cannot use the Internet?	0.64	0.65
χ^2^/*df*		2.13	1.53
RMSEA		0.09	0.06
CFI RSMR		0.78 0.08	0.90 0.06

Factor loadings related to Items 8 (Do you think you should use the Internet less often?) and 9 (Have you unsuccessfully tried to spend less time on the Internet?) were low, indicating a small relation to the total score.

### Criteria validity

Using the Enter method, the multiple linear regression resulted in a significant model (F_(7,145) _= 9.66 and p < 0.0005), which explained about 29% of the total variance. From the standardized residual plot, two outliers were identified (a 15-year-old boy with a total CIUS score of 2 compared with a predicted value of 25 and a 17-year-old girl with a total CIUS score of 5 and a predicted value of 29). Although lognormal transformations were used on the dependent variable to remedy for the skewness of the distribution, this did not solve the problem. It was then decided to discard them. The new regression still resulted in a significant model (F_7,143 _= 12.83 and p < 0.0005) and improved the explained variance (R^2^_adj _= 35.6%) without much changing either the previous regression coefficients or their sign. Only one regressor, "daily hours spent online," was statistically significant, whereas the other independent variables were not significant predictors of Internet addiction (Table 3).

**Table 3:** Results of linear Regression Coefficients^a^

*Model*	*Unstandardized coefficients*		*Standardized coefficients*			*95% CI for B*	
	
	B	Std. Error	Beta	*t * ^*1*^	Sig.	Lower bound	Upper bound
(Constant)	20.83	4.55		4.58	< 0.0005	11.836	29.823
Age	-0.18	0.24	-0.06	-0.75	0.46	-0.65	0.29
Sex	-0.07	1.1	-0.01	-0.07	0.95	-2.12	2.27
Hours spent online daily	2.42	0.35	0.57	6.88	< 0.0005	1.72	3.11
Internet used for:							
- less than 1 year	Ref						
- 1 to 3 years	-3.49	1.77	-0.20	-1.97	0.051	-7	0.01
- 3 to 5 years	-2.66	1.71	-0.17	-1.55	0.12	-6.06	0.73
- 5 years or more	-2.56	2.00	-0.14	-1.28	0.20	-6.52	1.40

^a^Dependent variable: CIUS total score. The number of degrees of freedom (df) associated with the t-test is n - p - 1 = 143.

## Discussion

The present study examined the psychometric properties of the Arab version of the CIUS. The main result of the study is that the CFA indicates that a one-factor model of the CIUS has good psychometric properties and fits the data well.

The small loadings found for Items 8 and 9, two items specifically related to the perspective of possible behavior change (*Do you think you should use the Internet less often*? *Have you unsuccessfully tried to spend less time on the Internet*?), were possibly due to a more precontemplative stage (considering excessive Internet use as not a problem) [39] of the present sample of Internet users and/or to a society that is more open to Internet use because it is still considered to be major progress.

In concordance with the findings of Meerkerk et al. [22], a significant positive association was found between CIUS scores and the daily duration of Internet use [22]. In this sample, mainly composed of young Arabic participants, no significant relationship was found between CIUS scores and gender. Nevertheless, previous studies found heterogeneous findings concerning gender differences in problematic Internet use, although men were generally found to be more addicted to the Internet than were women [15,19]. It is difficult to draw more conclusions from the study at hand because of the sample size and the lack of information related to possible intermediate variables such as psychosocial factors (i.e., motivations to go online, impulsivity, or escapism) [11,40] or the Internet applications used.

The main limitations of the present study are in the sampling of participants, who were young (average age 17.6 ± 2.9 years old) and not an overall addictive sample, and in the lack of complementary measures such as psychosocial outcomes to test the convergent and divergent validity of the scale.

Despite these limitations, our findings emphasize that the Arabic version of the CIUS is a valid self-report to measure problematic Internet use.

## Consent

Written informed consent was obtained from the patients for publication of this report and accompanying images. A copy of the written consent is available for review by the Editor-in-Chief of this journal.

## Competing interests

The authors declare that they have no competing interests.

## Authors' contributions

YK designed the study. AC conducted the statistical analyses. KA conducted the recruitment procedure. YK and KA contributed to the scale translation procedure. YK and JB wrote the first draft and compiled the co-authors' suggestions. All authors participated in the drafting of the manuscript and approved the final version.

## Supplementary Material

Additional file 1 **Compulsive Internet Use Scale (CIUS)**. Arab-language translation by Khazaal et al. Click here for file
