# Supplementary material for: Arabic validation of the Compulsive Internet Use Scale (CIUS)
Source: Subst Abuse Treat Prev Policy. 2011 Nov 29;6:32. doi: 10.1186/1747-597X-6-32 (PMC3247020; doi:10.1186/1747-597X-6-32)
Supplement: Additional file 1 — Compulsive Internet Use Scale (CIUS). Arab-language translation by Khazaal et al. [file 1747-597X-6-32-S1.PDF]

COMPULSIVE INTERNET USE SCALE (CIUS)

CIUS [22]: Arab-language translation by Khazaal et al.

---

1. إلى أي درجة تجد صعوبة في التوقف عن استخدام الإنترنت، في حين كنت على الانترنت؟

- ❑ ابدا
- ❑ نادرا
- ❑ احيانا
- ❑ اكثر الاحيان
- ❑ غالبا

2. بأي كثرة تقرر استخدام الانترنت بالرغم من نيتك بالتوقف عن استخدامه

- ❑ ابدا
- ❑ نادرا
- ❑ احيانا
- ❑ اكثر الاحيان
- ❑ غالبا

3. إلى أي مدى ينصحك اهلك والاصدقأ بالتخفيف من استخدام الانترنت؟

- ❑ ابدا
- ❑ نادرا
- ❑ احيانا
- ❑ اكثر الاحيان
- ❑ غالبا

4. إلى أي مدى تفضل الجلوس وتصفح صفحات الانترنت بدل تمضية الوقت مع الآخرين؟

- ❑ ابدا
- ❑ نادرا
- ❑ احيانا
- ❑ اكثر الاحيان
- ❑ غالبا

5. إلى أي مدى تنقصك ساعات نوم بسبب الانترنت؟

- ❑ ابدا
- ❑ نادرا

- ❑ احيانا
- ❑ اكثر الاحيان
- ❑ غالبا

6. إلى أي مدى تفكر بالانترنت عندم تكون خارج المنزل؟
- ❑ ابدا
- ❑ نادرا
- ❑ احيانا
- ❑ اكثر الاحيان
- ❑ غالبا

7. إلى أي مدى تفرح لإستعمالك القادم للانترنت؟
- ❑ ابدا
- ❑ نادرا
- ❑ احيانا
- ❑ اكثر الاحيان
- ❑ غالبا

8. إلى أي مدى تفكر في تخفيف استخدام الانترنت؟
- ❑ ابدا
- ❑ نادرا
- ❑ احيانا
- ❑ اكثر الاحيان
- ❑ غالبا

9. *إلى أي مدى نجحت في تخفيف ساعات استخدام الانترنت؟
- ❑ ابدا
- ❑ نادرا
- ❑ احيانا
- ❑ اكثر الاحيان
- ❑ غالبا

10. الى اي مدى تنجز أعمالك بسرعة للذهاب وتصفح الانترنت؟
- ❑ ابدا
- ❑ نادرا
- ❑ احيانا
- ❑ اكثر الاحيان
- ❑ غالبا

11. إلى أي مدى تهمل واجبنك اليومية من فروض مدرسية وعملية ومنزلية لأنك تفضل الجلوس على الانترنت؟
- ❑ ابدا
- ❑ نادرا
- ❑ احيانا

- ❑ اكثر الاحيان
- ❑ غالبا

12. إلى اي مدى تلجأ الى الانترنت لدى انخفاض معنوياتك؟
- ❑ ابدا
- ❑ نادرا
- ❑ احيانا
- ❑ اكثر الاحيان
- ❑ غالبا

13. إلى أي مدى تستخدم الانترنت للهروب من الهموم او لمعالجة شعور سلبي؟
- ❑ ابدا
- ❑ نادرا
- ❑ احيانا
- ❑ اكثر الاحيان
- ❑ غالبا

14. إلى اي مدى تشعر أنك متوتر أومكبوت لعدم قدرتك على استخدام الانترنت؟
- ❑ ابدا
- ❑ نادرا
- ❑ احيانا
- ❑ اكثر الاحيان
- ❑ غالبا

*(reversed item)
